# Panax ginseng Extract Improves Follicular Development after Mouse Preantral Follicle 3D Culture

**DOI:** 10.22074/cellj.2019.5733

**Published:** 2019-02-25

**Authors:** Abbas Majdi Seghinsara, Hamed Shoorei, Mohammad Mehdi Hassanzadeh Taheri, Arash Khaki, Majid Shokoohi, Moloud Tahmasebi, Amir Afshin Khaki, Hossein Eyni, Sadegh Ghorbani, Khadijeh Riahi Rad, Hossein Kalarestaghi, Leila Roshangar

**Affiliations:** 1 Department of Anatomical Sciences, Faculty of Medicine, Tabriz University of Medical Sciences, Tabriz, Iran; 2 Department of Anatomical Sciences, Faculty of Medicine, Birjand University of Medical Sciences, Birjand, Iran; 3 Department of Pathology, Islamic Azad University, Tabriz Branch, Tabriz, Iran; 4 Department of Anatomical Sciences, Faculty of Medicine, Tarbiat Modares University, Tehran, Iran; 5 Department of Horticulture Science, Tarbiat Modares University, Tehran, Iran; 6 Research laboratory for Embryology and Stem Cells, Department of Anatomical Sciences and Pathology, School of Medicine, Ardabil University of Medical Sciences, Ardabil, Iran; 7 Stem Cell Research Center, Tabriz University of Medical Sciences, Tabriz, Iran

**Keywords:** Gene Expression, Ovarian Follicle, Panax ginseng, Steroid Hormone

## Abstract

**Objective:**

*Panax* ginseng is a popular traditional herb that has been used in complementary and alternative medicine in eastern Asia, and it possesses pharmacologically active compounds like ginsenosides (GSs). This study aimed to investigate the impact of *Panax* ginseng extract (PGE) at different concentrations on *in vitro* follicular function and development in a three-dimensional (3D) culture system fabricated using sodium alginate after 12 days of culture.

**Materials and Methods:**

In this experimental study, preantral follicles (n=661) were mechanically isolated from the ovaries of 14-day-old female NMRI mice using 29-gauge insulin syringes. Follicles were individually capsulated within sodium alginate, and divided into four groups including control and experimental groups 1, 2, and 3. Then, they were cultured for 12 days in the medium supplemented with different concentrations of PGE (0, 50, 100, and 500 µg/ mL, for control groups and groups 1, 2 and 3, respectively). At the end of the culture period, the mean diameter and maturation of follicles, follicular steroid production, mRNA expression level of proliferating cell nuclear antigen (PCNA) and follicle stimulating hormone receptor (FSH-R), and reactive oxygen species (ROS) levels in collected metaphase-II (MII) oocytes were determined.

**Results:**

The mean diameter of follicles in group 2 was significantly increased as compared to other groups (P<0.001). The percentages of the survival and maturation rate and levels of secreted hormones were higher in group 2 than the other groups (P<0.05). Follicles cultured in the presence of PGE 100 µg/mL had higher levels of proliferation cell nuclear antigen (PCNA) and follicle stimulating hormone receptor (FSH-R) mRNA expression in comparison to other groups (P<0.05). Moreover, oocytes collected from groups 2 and 3 had lower levels of ROS as compared to other groups (P<0.05).

**Conclusion:**

Our results suggest that PGE at the concentration of 100 µg/mL induces higher follicular function and development in the 3D culture system.

## Introduction

Folliculogenesis is a complex and dynamic process which has a key role in maintaining the continuity of mankind’s life (1-3). Folliculogenesis is regulated by several autocrine and paracrine factors (4). 

*In vitro* culture and maturation (IVC-IVM) of primordial and preantral follicles as well as IVM of cumulus-oocyte complexes (COCs) have been developed for investigating follicular growth and oocyte maturation mechanisms, and used as a source for *in vitro* fertilization (IVF) of the matured oocytes in assisted reproductive technology (ART) (5). Therefore, to investigate mechanisms underlying the folliculogenesis and explore the effects of factors such as antioxidant, hormones, and growth factors on the growth and maturation of follicles and oocytes, a myriad types of cell culture systems including two- and three-dimensional (3D) methods have been suggested and developed (4). 

In two-dimensional (2D) IVC system, isolated follicles (i.e. preantral) or granulosa cell (GC)-oocyte complexes grow in multi-well plates or dishes, then, GCs attach to the surface of culture vessel and migrate away from the oocyte (4). This system is not sufficient to sustain the normal architecture of follicles similar to that seen *in vivo*; therefore, follicles are unable to complete the maturation process because of partial loss of the oocyte follicle cells interactions (4, 6). On the other hand, in 3D IVC models, each isolated follicle is encapsulated in extracellular matrices (ECM), such as agar/agarose, hyaluronic acid (HA), alginate, collagen, and chitosan. Thus, encapsulated follicles not only do not attach to the bottom of the culture vessel, but also keep their spherical growth and native morphology. Moreover, in 3D systems, good interactions between the somatic cell and oocyte occur when optimized for the follicular growth and oocyte development (7). It has been suggested that, compared to other types of culture, gene expression profile of cells grown in a 3D culture system, is closer to that of cells grown *in vivo* (8). 

In each mentioned type of cell culture systems, since ovarian follicles are maintained in an incubator under higher concentrations of O_2_, the reactive oxygen species (ROS) are continuously produced (9). Overproduction of ROS can affect the IVC of preantral follicles because they can act as second messengers and lead to the opening of a nonspecific pore in the inner mitochondrial membrane, release of cytochrome c, and activation of caspase cascades ultimately resulting in apoptosis. Although overproduction of oxidative agents can cause cell damage and loss of follicular function, antioxidants can attenuate deleterious effects of oxidative stress (9, 10). 

Antioxidants are natural or man-made substances that bind free radicals and subsequently neutralize their destructive properties such as peroxidation of lipids and DNA breakage; hence, they can act as protective agents via scavenging free radicals (11, 12). Ginseng (*Panax ginseng*) is one of the most popular members of the family *Araliaceae* (13).

For thousands of years, ginseng has been used in traditional herbal medicine due to its pharmacological properties. It has been reported that ginseng root has various cellular activities, including anti-aging, anti- inflammatory, anti-tumor, and antioxidant effects. Several lines of evidence have reported GSs, polysaccharides, peptides, phenolic acids, phytosterols, tocopherols, policosanols, and fatty acids as the main components of ginseng, but recent pieces of evidence showed that GSs (ginseng saponins) are the major components of ginseng.

Ginsenosides are derived from dammarane-type triterpenoid with a steroid-like structure that consists of 30 carbon atoms and four trans-rings with a modified side chain at C-20. It has been reported that the molecular target of GSs is located not only in the cellular membrane but also inside the cell. Moreover, GSs can regulate cellular functions through non-genomic or the genomic pathways. In the non-genomic pathway, GSs are able to bind the membrane-associated receptors that can initiate the activation of the phosphorylation cascade and therefore, lead to the generation of so-called second messengers within the cell (13). Additionally, GSs by binding the intracellular nuclear hormone receptors, such as the proliferator-activated receptor, androgen receptor (AR), estrogen receptor (ER), and progesterone receptor (PR), can activate the genomic pathway.

*P. ginseng* has also been considered a phytoestrogenic herb that has potent estrogenic activity (14, 15). Phytoestrogens can exert estrogenic properties, directly through binding ERs (i.e. ERα or ß), which are mainly expressed in reproductive tissues or indirectly by activating ERs (13, 16). It has been reported that ERs mediate cellular effects of estrogens, as female steroid hormones mainly produced by the ovaries (13). One study found that *P. ginseng* could upregulate both ERa and ERß in the uterus and vagina. It could also enhance the serum level of estradiol in ovariectomized mice (15). Another study reported that addition of GSs to the culture media promoted the proliferation of cultured chicken ovarian germ cells (17). 

However, the impact of *P. ginseng* extract (PGE) on the growth and maturation of preantral follicles in vitro, remains unknown. Therefore, the main purpose of the present study was to investigate the effects of different doses of PGE on the development and function of follicles cultured for 12 days by assessment of follicular growth, the rates of antrum formation and oocyte maturation, hormonal production, and ROS level in collected MII oocytes, as well as evaluation of the mRNA expression levels of follicle stimulating hormone receptor (*FSH-R*) and proliferation cell nuclear antigen (*PCNA*). 

## Materials and Methods

### Animals and collection of ovarian follicles

In this experimental study, 14 day-old female NMRI mice (n=100) were used. Animals were maintained in the laboratory animals of the Tabriz University of Medical Sciences under standard conditions (temperature 22 ± 2°C, humidity 55 ± 2%, and 12 hours/12 hours light/dark cycle). To remove mice ovaries, animals were sacrificed by dislocating the cervical vertebrae, then their bilateral ovaries were immediately dissected from oviducts, connective tissues (mesentery), and fat by 29-gauge insulin needles and transferred to dissection medium containing alpha-minimum essential medium (a-MEM, WelGENE, Korea) supplemented with antibiotics and 5% fetal bovine serum (FBS, Gibco, UK). The Ethical Committee of Tabriz University of Medical Sciences, Iran approved the present study (IR.TBZMED.REC.1396.555). 

### Study design

Under a stereomicroscope (Olympus, Japan), intact preantral follicles (n=661) of 150-160 micrometer (µm) in diameter, were mechanically isolated from immature mice ovaries using 29-gauge insulin syringes, and immediately collected by a micropipette. Then, only those with more centrally-located spherical oocytes which were surrounded by two or three layers of GCs and no apparent sign of necrosis were randomly divided into one control group and three experimental groups. The control group experimental groups 1 (Exp. 1), 2 (Exp. 2), and 3 (Exp. 3) were respectively treated with 50, 100, and 500 µg/mL of P. ginseng extract (G115 or PGE) (commercially obtained from Pharmaton Company, Switzerland). One study quantified GSs content of the PGE powder (Pharmaton Company, Switzerland) and found Rg1 (4.61 ± 0.43 mg/g), Rb1 (1.39 ± 0.12 mg/g), Rb2 (11.59 ± 1.30 mg/g), Rf (2.36 ± 0.25 mg/g), Rd (9.06 ± 1.05 mg/g), Rc (3.99 ± 0.20 mg/g), and Re (9.59 ± 0.85 mg/g) in PGE (18). In this study, the base of culture medium of follicles for all groups was composed of a- MEM supplemented with 0.33 mM sodium pyruvate, 1% insulin, transferrin, and sodium selenite (ITS, Gibco, UK), 100 mIU/mL rFSH (Gonal-f, Switzerland), antibiotics (100 IU/ mL penicillin and 50 mg/mL streptomycin), and 5% FBS. Moreover, in the present study, PGE (10 g/mL) was prepared in dimethylsulfoxide (DMSO, Sigma-Aldrich, Germany), then diluted with media to provide different concentrations of PGE (i.e. 50, 100, and 500 µg/mL) for in vitro ovarian follicle culture. Furthermore, in this study, all experiments performed at least in three replicates. 

### *In vitro* three-dimensional culture system 

Three-dimensional culture system could increase follicular growth, GC differentiation, somatic cell proliferation, oocyte growth, and hormone production by maintaining cell-cell communication and paracrine signalling between the follicular cells and oocytes that eventually promote the growth of both cell types (4). In this stage, isolated follicles were encapsulated in sodium alginate (Sigma-Aldrich, Germany). In brief, sodium alginate was dissolved in deionized water to reach the concentration of 1% (w/v). Then, it was mixed with activated charcoal (0.5 g charcoal was added to 1 g sodium alginate) to improve alginate purity and also remove organic impurities. After charcoal treatment, sodium alginate solution was centrifuged at 5000 rpm for 5 minutes; then, it was passed through 0.22 µm filters (Millipore Filtration, Sigma-Aldrich, Germany). At the end of the process, aliquots of sodium alginate and charcoal- stripped were diluted with 1 X sterilized f phosphate buffer saline (PBS) to reach the concentration of 0.5% (w/v). Then, each isolated follicle was transferred to 10 mL of alginate droplet. Droplets were slowly falling into the chemical cross-linking solution (140 mM NaCl and 50 mM CaCl_2_) and left for 2-3 minutes (4). Each alginate droplet was removed and washed in a-MEM media, then transferred into 35-mm Petri dishes (SPL Life Science, Korea), which had been filled with 2535 droplets of culture medium (the volume of each droplet was 50 mL) overlaid together with mineral oil (Sigma-Aldrich, Germany). All capsulated follicles were cultured for 12 days under standard conditions (at 37.with 5% CO_2_).

### Morphological and diametrical assessment of follicles

During the culture period, the morphology of follicles was checked using an inverted microscope (Olympus, Japan) and of follicles photographs were taken at ×100 magnification. Follicles with extrusion of denuded oocytes or darkened oocytes, a disorganized arrangement of GCs, and darkening were considered “degenerated follicles”. To measure the diameter of follicles, the photographs were imported into ImageJ Software (http://rsbweb.nih.gov/ij) (n=30/each group). After calibration of ImageJ Software, the mean diameter of each follicle was calculated as the mean length of two perpendicular axes. 

### Induction of *in vitro* ovulation 

For induction of in vitro ovulation and oocyte maturation, 1.5 IU/mL human chorionic gonadotropin (hCG, Organon, Netherlands) on day 12 of culture, was added to the culture media. About 24 hours after adding HCG to culture media, the oocytes were scored as a germinal vesicle (GV), GV breakdown (GVBD), and metaphase II (MII) stages based on the appearance of the GV or polar body in the perivitelline space. 

### Assessment of steroid hormones 

The levels of hormones including 17-ß estradiol (E2), progesterone (P4), as well as dehydroepiandrosterone (DHEA) were measured by a Microplate Enzyme Immunoassay kit (sensitivity=6.5 pg/mL, Monobind Inc., USA), an enzyme-linked immunosorbent (ELISA) assay kit (sensitivity=0.1 ng/mL, DiaPlus Inc., USA), and a Microplate Enzyme Immunoassay kit (sensitivity=0.04 mg/mL, Monobind Inc., USA), respectively. On the last day of culture, half of the culture media was collected.

### RNA extraction, cDNA synthesis for molecular assessment and real-time quantitative reverse transcription polymerase chain reaction 

To evaluate gene expression, on culture day 12, follicles in all of the studied groups were collected (15 follicles/ each replicate). Briefly, total RNA was extracted from each group using a TRIzol reagent extraction method (Invitrogen, Paisley, UK) according to the manufacturer’s instructions. To eliminate any genomic DNA contamination, DNase I treatment was performed after RNA extraction using the RNeasy Micro kit (Invitrogen Life Technologies, Carlsbad, CA, USA). Then, RNA concentration was determined by spectrophotometry and adjusted to a concentration of 600 ng/mL. Subsequently, the cDNA was synthesized by a commercial kit (Thermo Scientific, EU) using oligo dT and reverse transcriptase according to the manufacturer’s instructions. Then, cDNA synthesis reaction which was performed for 60 minutes at 42°C, was terminated by heating for 5 minutes at 70°C. The obtained cDNA was stored at -70°C until use. Sequences of specific primers for *PCNA, FSH-R,* and glyceraldehyde-3-phosphate dehydrogenase (*GAPDH*) genes are shown in Table 1 (4). *GAPDH* gene was used as an internal control. The RT-qPCR was performed on Applied Biosystems (UK) according to the manufacturer’s instructions in a 48-well plate using 20 mL reaction volume consisting of 7 mL RNase/DNase free water, 1 mL forward primer, 1 mL reverse primer, and 10 mL SYBR Green Master Mix (Sigma-Aldrich, Germany). The real- time thermal condition included a holding step (the initial denaturation) of 1 cycle at 95°C for 10 minutes, a cycling step of 40 cycles of 95°C for 15 seconds, 58°C for 30 seconds and 72°C for 30 seconds, and the final extension step (the melt curve step) at 95°C for 15 seconds, 60°C for 1 minute, and 95°C for 15 seconds. Then, relative quantitative analysis of target genes was done by Pfaffl method (i.e. 2^-ΔΔCT^, ΔΔCT = ΔCt_Sample_-ΔCt_Control_).

### Reactive oxygen species assay 

The levels of ROS in the obtained MII oocytes after HCG treatment (n=10 for each group) were measured based on the method reported in our pervious study (4). Briefly, in the first stage that was done in a dark room, 10 pooled MII oocytes were incubated in assay buffer (40 mmol/L Tris-HCl, pH=7 at 37°C) containing 5 mmol/L 2’, 7’-dichlorodihydrofluorescein diacetate (Sigma-Aldrich, Germany) for 25 minutes. In the second stage, incubated oocytes were washed with PBS and sonicated at 50W for 3 minutes, then immediately centrifuged at 3000 rpm for 12 minutes at 4°C. Finally, the supernatants were collected and monitored by a spectrofluorimetric method at excitation and emission wavelengths of 480 and 520 nm, respectively (4). Data related to the ROS levels are presented as mM H_2_O_2_. 

### Statistical analysis 

Data were analyzed by SPSS version 22 software (SPSS Inc., USA). The results of different groups were compared using one-way ANOVA followed by Tukey post-hoc test and expressed as mean ± SD. Differences were considered statistically significant when the P<0.05. 

## Results

### Effect of *P. Ginseng* extract on the diameter of cultured isolated preantral follicles 

**Fig.1:**
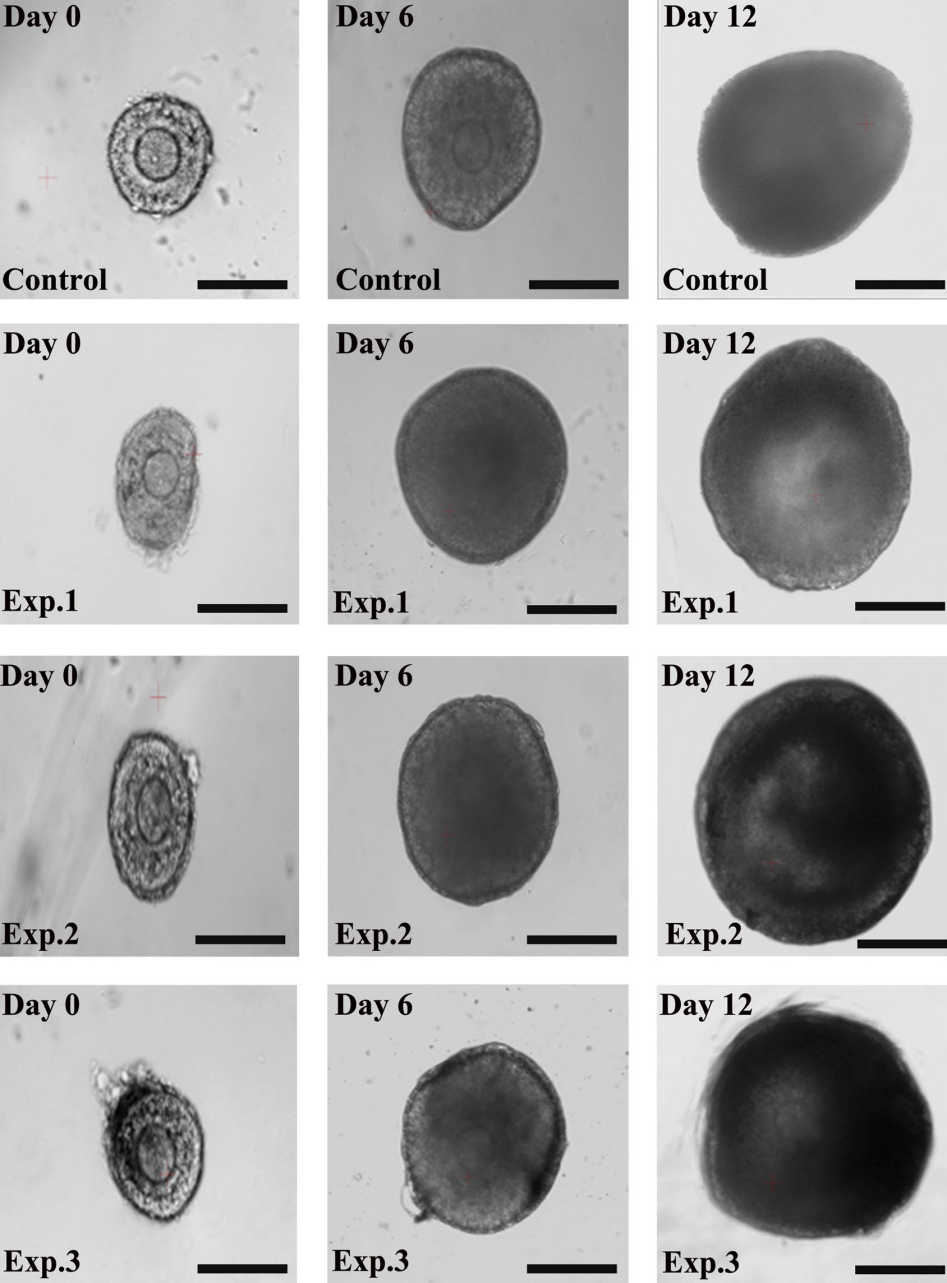
Images of the inverted microscope during in vitro three-dimensional follicular development in the alginate droplet on day 0 (first column), day 6 (second column), and day 12 (third column) in different groups. The control group containing 10% fetal bovine serum (FBS) without Panax ginseng extract (PGE), Exp.1, (group 1) that was treated with 50 µg/ml PGE, Exp.2, (group 2) that was treated with 100 µg/ml PGE, and Exp.3, (group 3) that was treated with 500 µg/ml PGE (scale bar: 100 µm).

**Fig.2:**
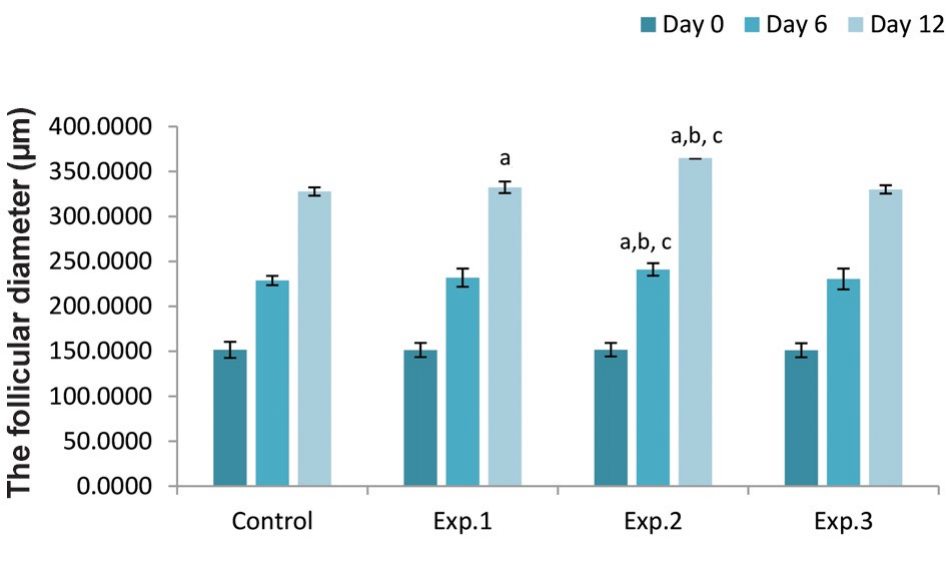
The average diameter of isolated preantral follicles (µm) during in vitro three-dimensional culture. a, b, and c show significant differences compared to control group (P<0.05), group 1 (P<0.001), and group 3 (P<0.001), respectively. Values are given as mean ± SD. Exp; Exprerimental group.

One of the objectives of this study was to investigate the impact of different concentrations of *P. ginseng* extract on the growth of isolated preantral follicles cultured in 0.5% alginate hydrogels for 12 days. In this regard, Figure 1 shows an invert micrograph of isolated cultured follicles. At the beginning of culture, the mean diameter of follicles was 151.48 ± 7 µm for all studied groups; however, during 12-day IVC period, in all of the studied groups, follicles increased in size and GC layers expanded (Fig .2). On the other hand, on days 6 and 12 of the culture, the mean diameter of cultured follicles which were treated with PGE 100 µg/mL was significantly increased compared to other groups (P<0.001). Moreover, on day 12 of culture, the mean diameter of follicles was significantly increased in experimental group 1 compared to control group (P<0.001). 

**Table 1:** The characteristic of primer sequences used in real-time quantitative reverse transcription polymerase chain reaction assays


Genes	Primer sequence (5´-3´)	GenBank accession numbers	Product size (bp)

PCNA	F: AGGAGGCGGTAACCATAG	NM-011045	76
	R: ACTCTACAACAAGGGGCACATC		
FSH-R	F: CCAGGCTGAGTCGTAGCATC	NM-013523.3	79
	R: GGCGGCAAACCTCTGAACT		
GAPDH	F: GGAAAAGAGCCTAGGGCAT	NM-007393	64
	R: CTGCCTGACGGCCAGG		


**Table 2:** Developmental rates of isolated preantral follicles after 12-day in vitro culture


Groups	Number of follicles	Number of survived	Number of antrum formation	Number of MII

Control	149	105 (70.48 ± 0.7)	44 (41.9 ± 2.49)	26 (24.64 ± 2.38)
Exp.1	167	121 (72.55 ± 1.58)^a^	52 (42.8 ± 2.37)	32 (25.51 ± 1.98)
Exp.2	174	140 (80.45 ± 0.76)^a^^b^^c^	71 (50.78 ± 3.22)^a^^b^^c^	44 (31.48 ± 2.00)^a^^b^^c^
Exp.3	171	122 (71.49 ± 1.14)	52 (42.70 ± 1.33)	31 (25.39 ± 1.06)


The control group containing 10% fetal bovine serum (FBS) without Panax ginseng extract (PGE), Exp.1, (group 1) that was treated with 50 µg/ml PGE, Exp.2, (group 2) that was treated with 100 µg/ml PGE, and Exp.3, (group 3) that was treated with 500 µg/ml PGE. ^a, b^, and^ c^ show significant differences compared to the control group (P<0.05), group 1 (P≤0.001), and group 3 (P≤0.001), respectively. Values are given as mean ± SD.

### Effect of *P. Ginseng* extract on follicular developmental rate

The developmental rate of follicles, including survival rate, antrum formation, as well as MII rate, are summarized in Table 2. Although the survival rate of follicles was significantly increased in experimental group 1 (treated with PGE 50 µg/mL) in comparison to control group (P<0.05), the highest percentage of survival rate was observed in group 2 (treated with PGE 100 µg/ mL) compared to other groups (P≤0.001). Moreover, the highest percentages of antrum formation and MII rate were observed in group 2 compared to other groups (P≤0.001). 

### Effect of *P. ginseng* extract on hormonal productions of follicles 

To investigate the effects of PGE on hormonal productions of cultured follicles, on the last day of the culture i.e. day 12), the levels of steroid hormones namely, E2, P4, and DHEA in media collected from cultured follicles, were measured (Table 3). The levels of these hormones were significantly higher in the media of follicles cultured in the presence of PGE 100 µg/mL compared to other groups (P<0.05). Also, the hormonal productions were significantly higher in group 1 compared to the control group (P<0.05). Additionally, the level of E2 was significantly increased in group 3 in comparison to control group (P<0.05). On the other hand, no significant difference was observed in P4 and DHEA hormones levels between group 3 and control group. 

### Real-time quantitative reverse transcription polymerase chain reaction analysis

**Fig.3:**
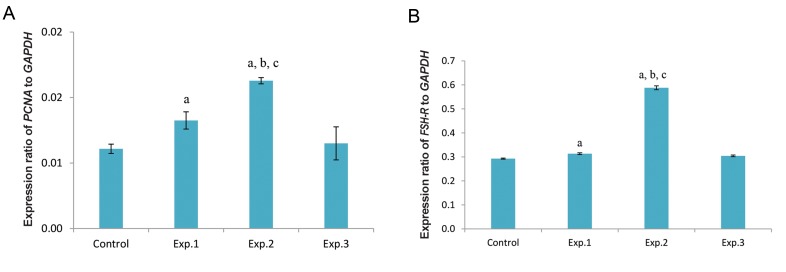
Effects of Panax ginseng extract on expression levels of PCNA and FSH-R mRNA on day 12 of culture as analyzed by real-time quantitative reverse transcription polymerase chain reaction (qRT-PCR). A. The mRNA expression of PCNA. a, b, and c show significant differences compared to control group (P<0.05), group 1 (P<0.05), and group 3 (P<0.001), respectively and B. The mRNA expression of FSH-R. a, b, and c show significant differences compared to control group (P<0.05), group 1 (P<0.001), and group 3 (P<0.001), respectively. Exp; Exprerimental group.

On the last day of culture, the effects of *P. ginseng* extract on the expression levels of *PCNA* and FSH-R mRNA were investigated by RT-qPCR (Fig .3A, B). The results showed increased expression of PCNA and *FSH-R* mRNA in group 1 when compared with control (P<0.05). Also, they were significantly higher in group 2 than the other groups (P<0.05). On the other hand, the relative expression of mentioned genes in group 3 was not significantly different from that of the control group. 

**Table 3:** The levels of steroid hormones (E2, P4, and dehydroepiandrosterone) in media collected on the last day of culture


Groups	17-ß estradiol (E2) (ng/ml)	Progesterone (P4) (ng/ml)	DHEA (μg/ml)

Control	1.99 ± 6.80	27.74 ± 2.09	22.47 ± 1.74
Exp.1	2.26 ± 6.30^a^	33.56 ± 2.25^a^	25.86 ± 2.11^a^
Exp.2	2.56 ± 4.54^a^^b^^c^	81.69 ± 1.65^a^^b^^c^	29.58 ± 1.12^a^^b^^c^
Exp.3	2.23 ± 6.30^a^	31.24 ± 1.41	23.19 ± 1.10


The control group containing 10% fetal bovine serum (FBS) without Panax ginseng extract (PGE), Exp.1, (group 1) that was treated with 50 µg/ml PGE, Exp.2, (group 2) that was treated with 100 µg/ml PGE, and Exp.3, (group 3) that was treated with 500 µg/ml PGE. ^a , b^, and ^c^ show significant differences compared to control group (P<0.05), group 1 (P<0.05), and group 3 (P<0.05), respectively. Values are given as mean ± SD. DHEA; Dehydroepiandrosterone.

### Effect of *P. Ginseng* extract on intracellular reactive oxygen species levels in collected metaphase-II oocytes

**Fig.4:**
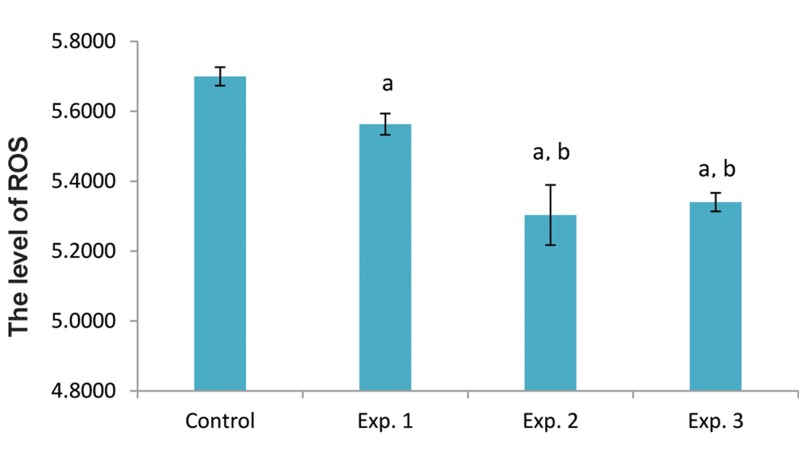
Reactive oxygen species (ROS) levels in different groups on the last day of culture (i.e. day 12). The control group containing 10% fetal bovine serum (FBS) without Panax ginseng extract (PGE), Exp.1, (group 1) that was treated with 50 µg/ml PGE, Exp.2, (group 2) that was treated with 100 µg/ml PGE, and Exp.3, (group 3) that was treated with 500 µg/ml PGE. a and b show significant differences compared to control group (P<0.05), and group 1 (P=0.002), respectively. Values are given as mean ± SD. Exp; Exprerimental group.

The antioxidant benefits of *P. ginseng* extract on MII oocytes, which were released after hCG treatment, were evaluated by measuring the abundance of intracellular ROS in oocytes using the spectrofluorimetric method (at excitation and emission wavelengths of 480 nm and 520 nm, respectively). The levels of ROS in collected MII oocytes in different groups are shown in Figure 4. Measuring the levels of ROS in collected MII oocytes revealed a significant difference between groups that received PGE and the control group (P<0.05). Also, there was a significant decrease in the levels of ROS of MII oocytes collected from groups 2 and 3 when compared to group 1 (P=0.002). Additionally, no significant difference was observed between groups 2 and 3 in this regard. 

## Discussion

This study investigated the impacts of different concentrations of *P. ginseng* extract on the growth and maturation of isolated preantral follicles after 12day culture in a 3D culture system fabricated using the sodium-alginate scaffold. 

However, during IVM of isolated preantral follicles, the average diameter of follicles was increased in all groups. But, follicles cultured in the media supplemented with PGE 100 µg/mL showed larger diameters compared to other groups. 

Moreover, our results showed that the follicles cultured in PGE 100 µg/mL -supplemented media had the highest follicular survival rate compared to other groups. Of note, in group 1, the survival rate was higher than that of the control group. Developmental rates namely the antrum formation and MII oocytes were significantly increased in group 2 (treated with PGE 100 µg/mL) compared to other groups. 

Also, in the medium containing PGE 100 µg/mL (group 2), production of steroid hormones by follicles (E2, P4, and DHEA) was higher than other groups. Moreover, the levels of steroid hormones in the media of group 1 (treated with PGE 50 µg/mL) were significantly increased in comparison to the control group. Consistently, the level of E2 in group 3 was significantly higher than that of the control group.

Furthermore, expression levels of *PCNA* and *FSH-R* mRNA were significantly increased in group 2 (treated with PGE 100 µg/mL).

Among the treated groups, follicles in the group (treated with PGE 100 µg/mL) had better growth and maturation compared to other groups. It seems the highest concentration of *ginseng* (500 µg/mL) may not be beneficial for *in vitro* ovarian follicle culture; hence, the growth and maturation of follicles were not improved as much as that observed in group 2 (treated with 100 µg/mL PGE). One study that investigated the antiviral effects of *Korean red ginseng* (KRG) at different concentrations (0, 5, 6.7, 10, 20 µg/mL), showed that the highest concentration (20 µg/mL) of KRG extract had cytotoxic effects on cell lines (19). Accordingly, it has been demonstrated that GSs are capable of binding directly to ERs or stimulating estrogenic responses independently of binding to ER. This phenomenon can enhance the estrogenic response even at low concentrations (20). However, the results of the present study showed that different concentrations of *P. ginseng* extract have different impacts on the growth and maturation of follicles. Hence, it appears that the effect of *ginseng* on *in vitro* growth and maturation of follicles and oocytes is dose- dependent and this extract acts as an anti-oxidant and anti-proliferative agent at doses of 50 and 100 µg/ mL. Since there was no sufficient information about the improvement of follicular growth and maturation by supplementing the culture media with PGE, we performed this research to examine the effect of this herbal extract. 

For several centuries, ginseng has been used as one of the most important herbal medicines in the East because of its diverse pharmacological effects on many systems, such as the immune system, central nervous system (CNS) and endocrine system (13, 17). Moreover, it has been reported that the major activecomponents of ginseng are GSs or ginseng saponins. Moreover, more than 100 different GSs with various pharmacological activities have been isolated fromthe root of *P. ginseng* (13, 21). Furthermore, the mostabundant GSs are Rb1, Rb2, Rg1, and Re (13). It has been proven that ginseng due to its antioxidant activity, enhances the function of reproductive organs (13). One study found that treatment with *Korean ginseng* saponins (GSs) modulated the process of steroidogenesis and improved the oocyte quality in the ovary of immature rats which were pre-treated with a super-ovulatory dose of pregnant mare serum gonadotropin (PMSG) (22). On the other hand, a clinical trial done on women experiencing menopausal disorders showed that intake of *Korean ginseng* powder improves the ovarian function via its estrogenic activity and increasing blood supply into the ovary (23). Another study reported that American ginseng could protect the ovary against premature ovarian failure (POF) by preventing the ovarian aging and regulating ovulation (24). Liu and Zhang (17) reported that adding GS at concentrations of 0.1, 1, and 10 µg/mL, could increase the number of chicken ovarian germ cells and elevate PCNA expression.

PCNA is a 36 kDa protein expressed in fetal and adult ovaries of several mammals and it is considered a marker for GCs proliferation (4). The crucial symbol of follicles development is the proliferation of GCs. It has been shown that GCs or oocytes of primordial follicles of rat, do not express PCNA; however, the expression of PCNA would be enhanced with the initiation of follicle growth (25). The higher PCNA expression has been considered a characteristic of actively growing follicles. Furthermore, it has been indicated that PCNA acts as a key factor in DNA replication and repair, the development of ovarian follicles as well as cell survival (4). Alternatively, although the mechanism underlying GS’s effects on follicular development and oocyte maturation is not fully elucidated, the meiotic maturation-induced effect of GSs on the oocytes of the mouse has been reported (26). Moreover, the pro- proliferation effects of GSs on diverse cells, such as ovarian germ cells, neurons and endothelial cells have been proven (17, 27). Several studies showed that GSs have a direct pro-proliferative effect on GCs of chicken pre-hierarchical follicles, chicken primordial germ cells and mouse spermatogonia through activating protein kinase C (PKC) (27-29). The PKC family has been implicated in the control of cell proliferation, apoptosis, differentiation and neuronal activity in many cell types; also, PKC signalling pathways promote the steroidogenesis in differentiated GCs of pre-ovulatory follicles (30). One study found that GSs enhance the proliferation of chicken GCs and development of chicken pre-hierarchical follicles in a dose-dependent manner through activating PKC signalling pathways involved in up-regulation of *cyclin E-CDK2* and *cyclin D1-CDK6* genes (27). Therefore, in our study, enhancement of PCNA expression at mRNA levels in follicles cultured in the presence of PGE 50 and 100 µg/mL may be due to the direct proliferative effect of *P. ginseng* extract on GCs in a dose-dependent manner.

Furthermore, our results showed that treatment with PGE 50 and 100 μg/mL increases the expression of FSH-R mRNA. In females, FSH and its cognate receptor (i.e. FSH-R), which are exclusively located on GCs, are essential for the ovarian function and fertility (4). FSH stimulates preantral follicles to grow and promote estradiol production in GCs and regulates the expression of *FSH-R* in GCs (4). FSH-R is a glycoprotein that belongs to the family of G proteincoupled receptors and is expressed in growing follicles (31). When the FSH binds its cognate receptor, it induces the follicular transition from preantral to antral follicles and promotes cytochrome P450. It can also increase the transcription of *CYP19A1* in GCs involved in converting theca cell-derived androgens namely, dehydroepiandrosterone and androstenedione into estrogens, estradiol, and estrone (32). It has been indicated that estrogens stimulate the proliferation of GCs and are absolutely necessary for the normal follicle growth (33). In a study that investigated the effects of ginsenoside Rg1 (one of the main compounds of PGE and a kind of natural estrogen) on POF induced by D-galactose (D-gal), it was shown that treatment with Rg1 could up-regulate the protein expression of FSH-R in GCs (34). Moreover, Lee et al. (35) reported that KRG could attenuate sub-chronic psychological stress-induced testicular damage and male sterility by modulating the proteins and mRNA expression levels of sex hormone receptors such as FSH-R, LH-R and AR. Therefore, it is suggested that PGE by up-regulating the expression level of FSH-R mRNA, could prevent the follicular regression and subsequently enhance the follicular development.

It has been reported that GSs act through steroid hormone receptors (36). In this regard, it has been indicated that KRG could upregulate the steroidogenic enzyme P450 (CYP11A1) in senescent rat testes (37). In females, in the estradiol biosynthesis pathways, cholesterol in the inner membrane of the mitochondria is converted into pregnenolone by CYP11A1 and then into estradiol by different pathways (38). Therefore, high concentrations of estradiol hormone in the media of cultured follicles supplemented with PGE 100 µg/ mL may stem from this phenomena. He et al. (34) reported that treatment with ginsenoside Rg1 could increase serum level of E2 in mice model of D-galinduced POF. Furthermore, high production of steroid hormones, P4 and DHEA, is associated with the activity of theca cells in the secretion of androgens through increasing the proliferation and differentiation of follicle cells, as well as increasing the aromatase activity in GCs numbers during IVC (39). 

In the present study, the level of ROS in oocytes was significantly decreased in groups 2 and 3 compared to other groups. It has been reported that Korean red ginseng oil prevents cell/tissue damages directly by scavenging ROS and inhibiting lipid peroxidation. The ability of PGE in reducing the level of ROS in oocytes is due to its antioxidant properties (40). 

## Conclusion

According to our results, *P. ginseng* extract exerts its effects on follicular development in a dose-dependent manner. Moreover, results of the present study demonstrated that P. ginseng extract 100 µg/mL not only increased the growth of isolated preantral follicles from the ovaries of pre-pubertal mice, but also enhanced their maturation rate after 12-day *in vitro* 3D culture. Further studies are warranted to illuminate the mechanism underlying the findings of the present experiment.
